# NFκB- and MAP-Kinase Signaling Contribute to the Activation of Murine Myeloid Dendritic Cells by a Flagellin A: Allergen Fusion Protein

**DOI:** 10.3390/cells8040355

**Published:** 2019-04-15

**Authors:** Tobias Moeller, Sonja Wolfheimer, Alexandra Goretzki, Stephan Scheurer, Stefan Schülke

**Affiliations:** Paul-Ehrlich-Institut, Vice President´s Research Group 1: Molecular Allergology, 63225 Langen, Hesse, Germany; tobias.moeller1990@gmx.de (T.M.); Sonja.Wolfheimer@pei.de (S.W.); Alexandra.Goretzki@pei.de (A.G.); Stephan.Scheurer@pei.de (S.S.)

**Keywords:** flagellin, TLR5, Bet v 1, birch allergy, vaccine, fusion protein, metabolism, MAPK, NFκB, signalling

## Abstract

Fusion proteins incorporating the TLR5-ligand flagellin are currently undergoing clinical trials as vaccine candidates for many diseases. We recently reported a flagellin:allergen fusion protein containing the TLR5-ligand flagellin A (FlaA) from *Listeria monocytogenes* and the major birch pollen allergen Bet v 1 (rFlaA:Betv1) to prevent allergic sensitization in an experimental mouse model. This study analyzes the signaling pathways contributing to rFlaA:Betv1-mediated pro- and anti-inflammatory cytokine secretion and cell metabolism in myeloid dendritic cells (mDCs) in vitro. The influence of mammalian target of rapamycin (mTOR)-, NFκB-, and MAP kinase (MAPK)-signaling on cytokine secretion and metabolic activity of bone marrow (BM)-derived mDCs stimulated with rFlaA:Betv1 were investigated by pre-treatment with either mTOR- (rapamycin), NFκB- (dexamethason, BMS-345541, TPCA-1, triptolide, or BAY-11) or MAPK- (SP600125, U0126, or SB202190) inhibitors, respectively. rFlaA:Betv1-mediated IL-10 secretion as well as activation of mDC metabolism, rather than pro-inflammatory cytokine secretion, were inhibited by rapamycin. Inhibition of NFκB-signaling suppressed rFlaA:Betv1-induced IL-12, while inhibition of MAPK-signaling dose-dependently suppressed rFlaA:Betv1-induced IL-10 as well as pro-inflammatory IL-6 and TNF-α production. Notably, with the exception of a partial JNK-dependency, rFlaA:Betv1-mediated effects on mDC metabolism were mostly NFκB- and MAPK-independent. Therefore, MAPK-mediated activation of both NFκB- and mTOR-signaling likely is a key pathway for the production of pro- and anti-inflammatory cytokines by flagellin fusion protein vaccines.

## 1. Introduction

The incidence of allergic diseases has steadily increased over the last 70 years, causing significant decreases in quality of life in affected patients and economic problems [1,2]. Besides symptomatic treatment or avoidance of the respective allergens, allergen specific immunotherapy (AIT) with allergen extracts is the only disease altering treatment option available so far. 

Currently, AIT is not convenient for patients due to a multi-year treatment regimen, only partially efficacious for some allergies, and can be hampered by unwanted side effects [3]. To improve AIT, novel vaccine candidates and accompanying adjuvants that increase efficacy while decreasing unwanted adverse-effects are needed [4]. In line with this, TLR-ligands with an intrinsic ability to induce robust innate immune responses are of special interest for their utilization as adjuvants. While for example the TLR4-ligand lipopolysaccharide (LPS), a very well characterized cell wall component of Gram-negative bacteria, has strong immune activating properties, its usage as an adjuvant is strongly limited due to its inherent toxicity [5]. Moreover, nucleic acid-based TLR-ligands such as CpG (TLR9), R848 (TLR7/8), or Poly I:C (TLR3), are effective immune stimulators but are limited in their clinical efficacy due to problems with both toxicity and stability in vivo [5].

Interestingly, vaccines adjuvanted with the TLR5-ligand flagellin [6], a bacterial motility protein forming the body of the bacterial flagellum, were reported to be safe and well tolerated in clinical trials [7,8]. In this context, flagellin was demonstrated to be an effective mucosal adjuvant mediating protective immune responses [9,10,11]. When using flagellin as an adjuvant one of the major advantages is its proteinaceous nature allowing for the efficient generation of fusion proteins of flagellin and the antigen of choice by recombinant DNA technology. The generated fusion proteins combine antigen and adjuvant (flagellin) into a single molecule, which results in the efficient targeting of antigens to and simultaneous activation of TLR5^+^ antigen presenting cells (APCs) [11].

Consequently, fusion proteins combining flagellin with different antigens were investigated for their clinical potential to treat different diseases including, among others: influenza [12,13,14], poxvirus [15], West-Nile-Virus [16], tetanus [17], and *Pseudomonas* infection [18]. Such fusion proteins are also investigated as vaccines for the treatment of IgE-mediated type I allergies [19,20,21,22]. Kitzmüller and colleagues recently described the enhanced immunogenicity, reduced allergenicity, and intrinsic adjuvant activity of flagellin C:Betv1 fusion proteins in human monocyte-derived DCs and T cells from allergic patients [19]. So far, all studies testing such flagellin-containing fusion proteins demonstrated that these vaccine candidates have superior immune activating potential compared to the mixture of flagellin and antigen [12,13,14,15,16,18,20,23,24], making them very interesting vaccine candidates for future human application.

Despite their well-described immune activating potential, the mechanisms by which flagellin fusion proteins modulate immune responses are less well understood. In line with results obtained by Kitzmüller et al. [19], we recently showed a fusion protein consisting of the TLR5-ligand flagellin FlaA from *Listeria monocytogenes* and the major birch pollen allergen Bet v 1 (rFlaA:Betv1) to display strong immune modulating properties both in vivo and in vitro, characterized by the induction of both pro- and anti-inflammatory cytokine secretion from murine myeloid dendritic cells (mDCs) as well as peripheral blood mononuclear cells (PBMCs) from birch allergic patients [22]. Here, the pronounced secretion of the anti-inflammatory cytokine IL-10 was shown to suppress allergen-specific TH1- and especially TH2-responses [22]. Mechanistically, we showed that the activation of mDC metabolism resulted in a predominant production of energy by a high rate of glycolysis and lactic acid fermentation known as the Warburg effect [25] mediated by an activation of mammalian target of rapamycin (mTOR). Moreover, evidence was provided that induction of anti-inflammatory IL-10 secretion by rFlaA:Betv1, but not pro-inflammatory cytokine secretion (IL-1β, TNF-α, or IL-6 ) in mDCs, was dose-dependently inhibited by rapamycin (a specific inhibitor of the mTOR1-complex) and therefore dependent on mTOR activation [22]. 

Interestingly, inhibition of rFlaA:Betv1-induced IL-10 secretion by rapamycin correlated with enhanced production of the TH1-promoting cytokine IL-12, suggesting these two cytokines to be inversely regulated [22].Taken together, these results showed that the immune-modulatory cytokine secretion induced by this vaccine candidate was linked to the activation of mDC metabolism [22].

While the engagement of mTOR in the rFlaA:Betv1-induced anti-inflammatory IL-10 secretion was elucidated, the mechanism by which such fusion proteins induce pro-inflammatory cytokine secretion remained unclear. Therefore, the aim of this study was to further investigate if MAP kinase- and NFκB-signaling contribute to rFlaA:Betv1-induced pro- and anti-inflammatory cytokine secretion as well as the activation of mDC metabolism. 

Mitogen-activated protein (MAP) kinases are ubiquitously expressed protein kinases that either auto-phosphorylate endogenous serine and threonine residues or phosphorylate their substrates [26]. MAP kinases (MAPK) regulate cell proliferation, stress responses, apoptosis, and the induction of immune responses [26]. In mammalian cells, MAP kinases belong to three MAPK pathways: the ERK1/2- (p42/44), the c-JUN N-terminal kinase 1, 2, and 3 (SAP/JNK-1/2/3), and the p-38 MAPK-pathway [26]. The induction of pro-inflammatory cytokines such as TNF-α, IL-1β, IL-2, but also anti-inflammatory IL-10 and therefore the type of immune responses elicited by pathogens can be triggered by the activation of MAPK-signaling [27,28]. 

Mammalian nuclear factor “kapa-light-enhancer” of activated B cells (NFκB) is a family of five inducible transcription factors [29]. Under normal conditions, the NFκB proteins are predominantly kept inactive by cytoplasmic association with inhibitory IκB (inhibitor of kappa B) proteins which inhibit NFκB-DNA-binding activity [30]. Activation of NFκB signaling by either pro-inflammatory cytokines, pathogen-associated molecular pattern (PAMP)-mediated activation of pattern recognition receptors (PRRs) of the innate immune system, T- and B-cell receptor signaling, and ligation of lymphocyte co-receptors [29,31,32] results in degradation of the inhibitory IκB proteins, the release of NFκB dimers, and their translocation into the nucleus where they activate pro-inflammatory gene transcription [30]. 

The aim of the present study was to further characterize the signaling pathways contributing to the induction of pro- and anti-inflammatory cytokine secretion in mDCs and their effect on the activation of mDC metabolism in vitro. Our results suggest a flagellin fusion protein-mediated activation of both NFκB- and MAPK-signaling to contribute to pro- and anti-inflammatory cytokine secretion while only JNK MAPK activation contributed to metabolic activation. 

## 2. Materials and Methods

### 2.1. Generation of Recombinant Proteins 

Recombinant flagellin A from *Listeria monocytogenes* (FlaA, Acc. No: NC_003210) was generated according to ref. [33], recombinant birch pollen allergen Bet v 1 (Acc. No: X15877.1) according to ref. [34]. The fusion protein of rFlaA and rBet v 1 (rFlaA:Betv1) was generated according to ref. [22] by cDNA fusion using the cDNAs of both rFlaA and rBet v 1 as templates. All proteins displayed a purity >99%, correct folding of secondary structure elements as determined by circular dichroism-spectroscopy, and an endotoxin content of 1.14 pg/µg protein (rFlaA), <0.48 pg/µg protein (rBet v 1), and 1.7 pg/µg protein (rFlaA:Betv1) respectively (data not shown).

### 2.2. Mice

BALB/c mice (the Jackson Laboratory, Bar Harbor, Maine, USA) were bred at the animal facility of the Paul-Ehrlich-Institut under specific pathogen-free conditions. All animal experiments were performed in compliance with the German animal protection law.

### 2.3. In Vitro Generation of Mouse Bone Marrow-Derived Dendritic Cells, Stimulation, and Flow Cytometry

Mouse myeloid dendritic cells (mDCs) were generated as described previously [33]. On day 8 mDCs were seeded at 5 × 10^5^ cells/mL in 24-well plates (Thermo Scientific, Dreieich, Germany) and stimulated with the indicated equimolar concentrations of rFlaA + rBet v 1, or rFlaA:Betv1 for 24 h to 72 h. Ten µg/mL LPS (L5886, Sigma Aldrich, Taufkirchen, Germany) served as positive control. Supernatants were analyzed for cytokine secretion by ELISA using BD OptEIA™ ELISA Sets (BD Biosciences, Heidelberg, Germany). The viability of mDC cultures was assessed by flow cytometry using an LSR II flow cytometer (BD Bioscience, Heidelberg, Germany). Data were analyzed using FlowJo V.7 (Treestar Inc., Ashland, OR, USA).

### 2.4. Inhibitors

For inhibition experiments mDCs were pre-incubated with the indicated amounts of rapamycin (mTOR inhibitor), BAY-11-7082 (irreversible inhibitor of TNF-α-induced IkB-α phosphorylation resulting in inactivation of NFkB), triptolide (NFκB inhibitor), dexamethason (NFκB and MAPK inhibitor), the IKK-β inhibitors TPCA-1 (Abcam, Cambridge, UK) and BMS-345541 (Abcam, Cambridge, UK), as well as the MAPK inhibitors SP600125 (JNK MAPK inhibitor, Invivogen, Toulouse, France), SB-202190 (p38α/β MAPK inhibitor, Invivogen, Toulouse, France), or U0126 (MEK1/2 MAPK inhibitor, Cell Signaling Technologies, Leiden, The Netherlands) for 90 min and subsequently stimulated with equimolar amounts of rFlaA + rBet v 1 or rFlaA:Betv1 for either 30 min (Western Blot), 24 h (ELISA and cytotoxicity), or 72 h (ELISA and analysis of cell metabolic state). Toxicity of the used inhibitors was determined using the fixable viability dye eFlour780 (Thermo Fisher Scientific, Darmstadt, Germany, Repository Figure S1). Inhibitor concentrations showing toxic effects were excluded from the analysis. 

### 2.5. SDS-PAGE and Western Blot

SDS-PAGE was performed according to the method described by Laemmli [35] (cross linker c = 5%, total bis/acrylamid 15%) under reducing conditions. For Western Blot experiments mDCs were starved in RPMI1640 + 10% FCS (Sigma-Aldrich, Taufkirchen, Germany) with or without the indicated inhibitor concentrations and cultured for 3 h at 37 °C, 5% CO_2_ in either T25/75 flasks or FACS tubes. Subsequently, 1 × 10^6^ mDCs were stimulated for 30 min in RPMI1640 with the indicated proteins. 30 min post stimulation, cells were washed with ice cold PBS and subsequently lysed with 200 µL lysis buffer (62.5 mM Tris-HCl, pH 6.8 at 25 °C), 2% *w*/*v* SDS, 10% glycerol, 50 mM DTT, 0.01% *w*/*v* bromophenol blue) for 10 min on ice. Target proteins in lysates were detected by Western Blot using the iBind System (Thermo Fisher Scientific, Darmstadt, Germany) and antibodies from Cell Signaling Technologies (Leiden, The Netherlands): mTOR regulation antibody sampler kit, mTOR substrates antibody sampler kit, NFκB pathway sampler kit, MAPK family antibody sampler kit, loading control antibody sampler kit (HRP Conjugate). All grouped Western Blots were generated from the same experiment (one experiment for the left and one experiment for the right half of Figure 6) without stripping of the membranes (independent confirmatory experiments were performed under the same experimental conditions and detected either separately or in parallel). Molecular weights of the detected proteins were: Histone H3: 17 kDa, GAPDH: 37 kDa, phospho p38 MAPK: 43 kDa, phospho SAP/JNK MAPK: 46.5 kDa, phospho p42/44 ERK MAPK: 42/44kDa, phospho NFκBp65: 65 kDa, IκB-alpha: 40 kDa, phospho IκB-alpha: 40 kDa, phospho p70S6 kinase: 70 kDa; Detection was performed using AceGlowTM substrate (VWR, Darmstadt, Germany) and a Fusion-Fx7 Spectra (Vilber Lourmat, Eberhardzell, Germany). Band intensities in Western Blots were analyzed using ImageJ (imagej.nih.gov) as RLU normalized to unstimulated controls.

### 2.6. Analysis of Cell Metabolic State

The Warburg effect in stimulated mDC cultures was either assessed by visual examination or determined photometrically 72 h post stimulation by quantifying the optical density (OD) at 570 nm and calculating the Warburg effect as 1/OD570 normalized to unstimulated controls. Glucose concentrations in culture supernatants were determined 72 h post-stimulation using the Glucose (GO) Assay Kit (Sigma-Aldrich, Taufkirchen, Germany). The metabolic rate was derived from the measured glucose concentrations by calculating the glucose consumption in percent of medium without mDCs (glucose conc. in RPMI1640 = 2 mg/mL).

### 2.7. Statistical Analysis

Statistical analysis was performed using GraphPad Prism v6 to v8 for Mac or Windows (GraphPad Software, San Diego, CA, USA) using 2-way ANOVA tests with confidence intervals adjusted for multiple comparisons according to either Bonferroni or Turkey. For statistically significant results the following convention was used: * *p* < 0.05, ** *p* < 0.01, *** *p* < 0.001.

## 3. Results

### 3.1. rFlaA:Betv1-Induced Activation of mDC Metabolism and IL-10 Secretion is Mediated by an Activation of the mTOR1 Complex, Whereas Pro-Inflammatory Cytokine Secretion Is Mostly mTOR-Independent

**Figure 1:**
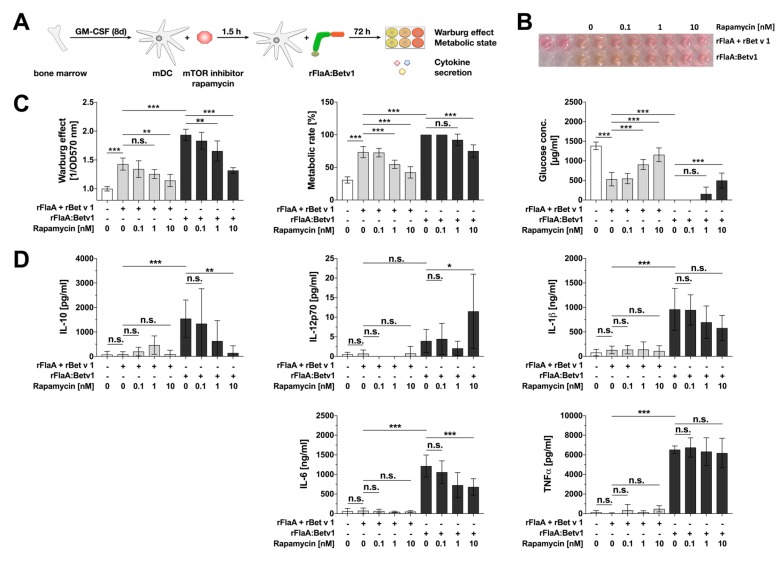
rFlaA:Betv1 induces a mTOR-dependent activation of mDC metabolism and IL-10 secretion. 0.5 × 10^6^ BALB/c mDCs were pre-treated with the indicated concentrations of rapamycin for 90 min and subsequently stimulated with either 17.4 µg rFlaA + 10 µg rBet v 1 or 27.4 µg rFlaA:Betv1 (all equimolar to each other) for another 72 h. Experimental setup (**A**), photographic documentation of the induced Warburg effect (**B**), and analysis of cell metabolic state (**C**). Cytokine secretion into cell supernatants was determined 72 h post stimulation by ELISA (**D**). Data are mean results of three independent experiments ± SD (**C** + **D**) or representative results taken from one out of three independent experiments (**B**). For statistically significant results the following convention was used: n.s.—not significant, * *p* < 0.05, ** *p* < 0.01, *** *p* < 0.001.

As reported before [22] (but with optimized cell numbers and additionally measured cytokines) stimulation of mDCs with rFlaA:Betv1 resulted in a significantly increased Warburg effect, metabolic rate, and glucose consumption which could dose-dependently be inhibited by pre-treatment of mDCs with rapamycin, a specific inhibitor of the mTOR1-complex (Figure 1A–C). Here, the activation of mDC metabolism upon stimulation with the mixture of rFlaA + rBet v 1 was significantly lower than with the rFlaA:Betv1 fusion protein (Figure 1A–C). 

In line with the strong activation of mDC metabolism, rFlaA:Betv1 stimulation resulted in a significantly increased secretion of both pro- (IL-1β, IL-6, and TNF-α) and anti-inflammatory (IL-10) cytokines from mDC cultures (Figure 1D). Here, rFlaA:Betv1-induced IL-10 secretion was dose-dependently inhibited by pre-treatment with rapamycin, while IL-12 secretion was slightly increased for the highest concentration of rapamycin only (Figure 1D). In accordance with previous results other rFlaA:Betv1-induced pro-inflammatory cytokine secretion was either not (TNF-α) or less (IL-1β, IL-6) affected by rapamycin pre-treatment and considered to depend less on mTOR activation than the observed IL-10 secretion (Figure 1D). Therefore, the pathways contributing to rFlaA:Betv1-induced pro- and anti-inflammatory cytokine secretion from mouse mDCs were further elucidated.

### 3.2. NFκB Inhibition Suppresses rFlaA:Betv1-Induced IL-12 Secretion while Enhancing IL-1β Secretion

**Figure 2:**
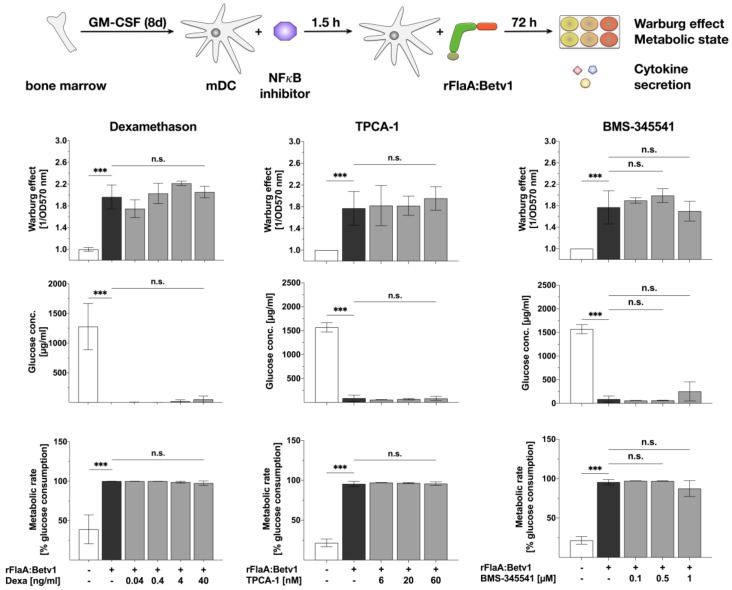
NFκB-signaling does not contribute to rFlaA:Betv1-induced activation of mDC metabolism: 0.5 × 10^6^ BALB/c mDCs were pre-treated with the indicated NFκB- (dexamethason) or IKBα- (TPCA-1 & BMS-345541) inhibitor concentrations for 90 min and subsequently stimulated with 27.4 µg rFlaA:Betv1 for another 72 h. The metabolic state of the stimulated mDCs was determined 72 h post-stimulation. Data are mean results of three independent experiments ± SD. For statistically significant results the following convention was used: n.s.—not significant, * *p* < 0.05, ** *p* < 0.01, *** *p* < 0.001.

**Figure 3:**
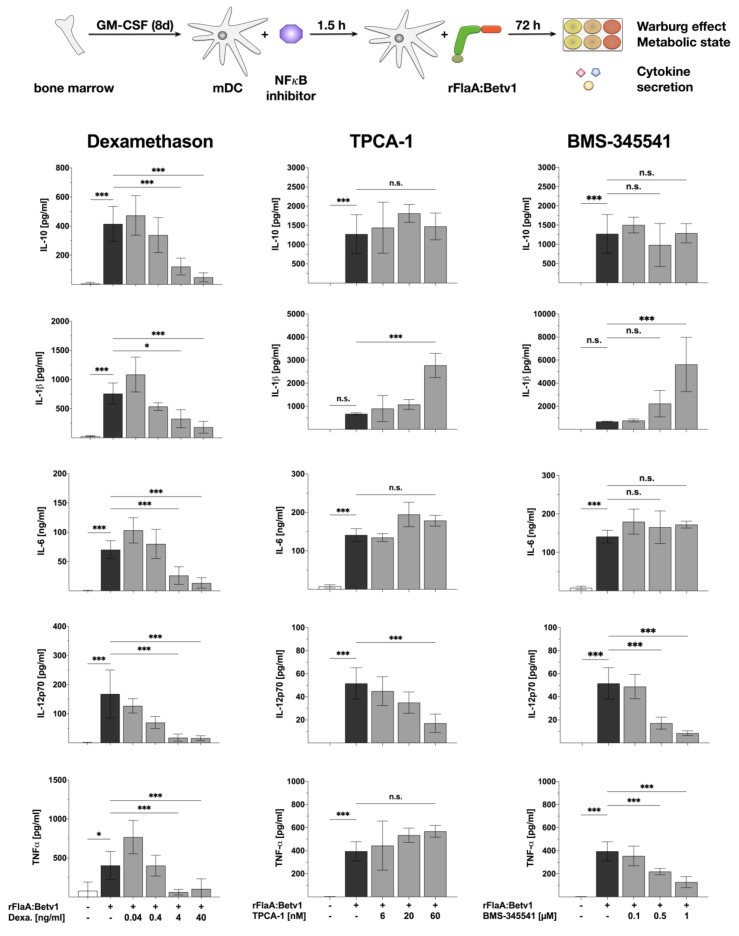
NFκB-signaling contributes to rFlaA:Betv1-induced IL-12p70 secretion in mDCs: 0.5 × 10^6^ BALB/c mDCs were pre-treated with the indicated NFκB- (dexamethason) or IKBα- (TPCA-1 & BMS-345541) inhibitor concentrations for 90 min and subsequently stimulated with 27.4 µg rFlaA:Betv1 for another 72 h. Cytokine secretion into cell supernatants was determined 72 h post-stimulation by ELISA. Data are mean results of three independent experiments ± SD. For statistically significant results the following convention was used: n.s.—not significant, * *p* < 0.05, ** *p* < 0.01, *** *p* < 0.001.

Next we further analyzed the intracellular signaling cascades contributing to rFlaA:Betv1-induced mDC activation. First we checked the contribution of NFκB-signaling to the rFlaA:Betv1-induced activation of mDC metabolism as well as the production of pro- and anti-inflammatory cytokines (Figure 2 and Figure 3) by pre-treating mDCs with either dexamethason (an agonist of the glucocorticoid receptor, indirectly inhibiting both NFκB- and MAPK-signaling), two different IKK-β inhibitors TPCA-1 and BMS-345541, triptolide (a NFκB-inhibitor, Repository Figure S2), or BAY-11 (an irreversible inhibitor of TNF-α-induced, non-canonical IκB-α phosphorylation, Repository Figure S3).

Here, dexamethason had no effect on rFlaA:Betv1-induced Warburg effect and glucose consumption from the culture medium (Figure 2), but dose-dependently and significantly inhibited the production of all investigated cytokines (Figure 3). 

Moreover, neither TPCA-1 nor BMS-345541 had any effect on rFlaA:Betv1 induced activation of mDC metabolism (Figure 2), but both inhibitors dose-dependently and significantly suppressed rFlaA:Betv1-induced IL-12p70 production while enhancing IL-1β secretion (Figure 3). While both inhibitors had no influence on rFlaA:Betv1-induced IL-10 and IL-6 production, TNF-α production was only inhibited by pre-treatment with BMS-345541, but not TPCA-1 (Figure 3).

Moreover, pre-treatment with the NFκB inhibitor triptolide significantly reduced the rFlaA:Betv1-induced Warburg effect by 32.2% and reduced the metabolic rate by 38.3% due to a considerable reduction in glucose consumption (Repository Figure S2). Triptolide pre-treatment dose-dependently suppressed rFlaA:Betv1-induced IL-10, IL-6, and TNF-α secretion while enhancing IL-12p70 production and having no effect on IL-1β production (Repository Figure S2). Pre-treatment of the mDCs with the used concentrations of BAY-11 had no effect on either rFlaA:Betv1-induced activation of mDC metabolism or cytokine secretion; except some evidence for reduction of IL-6 (Repository Figure S3). Toxic effects of the used inhibitor on mDC cultures were excluded by viability staining (Repository Figure S1).

Taken together, the results from the different inhibitors suggest that rFlaA:Betv1-induced NFκB-signaling contributes to the induction of IL-12 production while having little effect on mDC metabolism, IL-10, and IL-6 production.

### 3.3. MAPK Signalling Contributes to Both rFlaABetv1-Induced Pro- and Anti-Inflammatory Cytokine Secretion

**Figure 4:**
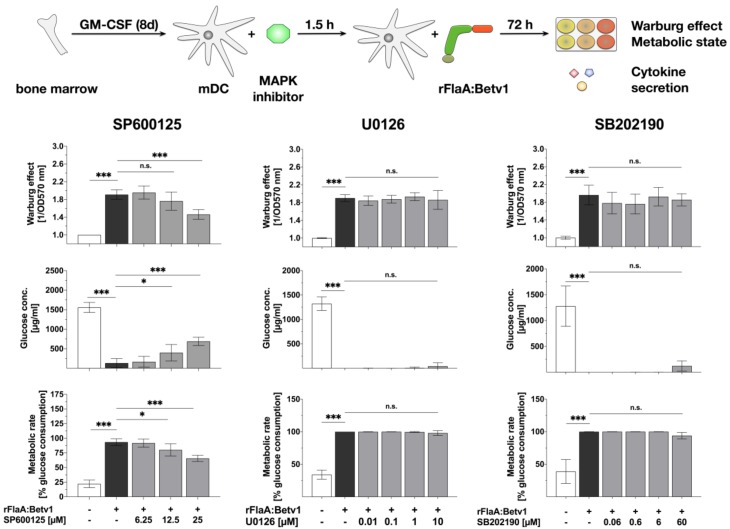
JNK MAP kinase-signaling contributes to rFlaA:Betv1-induced activation of mDC metabolism: 0.5 × 10^6^ BALB/c mDCs were pre-treated with the indicated MAPK inhibitor concentrations for 90 min and subsequently stimulated with 27.4 µg rFlaA:Betv1 for another 72 h. The metabolic state of the stimulated mDCs was determined 72 h post-stimulation. Data are mean results of three independent experiments ± SD. For statistically significant results the following convention was used: n.s.—not significant, * *p* < 0.05, ** *p* < 0.01, *** *p* < 0.001.

**Figure 5:**
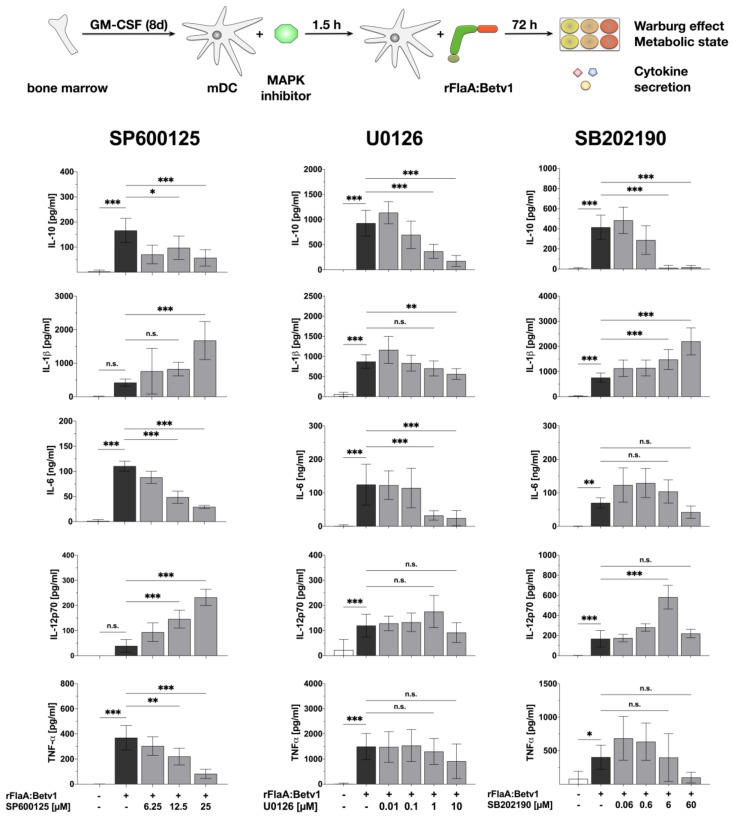
MAP kinase-signaling contributes to both rFlaA:Betv1-induced pro- and anti-inflammatory cytokine secretion in mDCs: 0.5 × 10^6^ BALB/c mDCs were pre-treated with the indicated inhibitor concentrations for 90 min and subsequently stimulated with 27.4 µg rFlaA:Betv1 for another 72 h. Cytokine secretion into cell supernatants was determined 72 h post-stimulation by ELISA. Data are mean results of three independent experiments ± SD. For statistically significant results the following convention was used: n.s.—not significant, * *p* < 0.05, ** *p* < 0.01, *** *p* < 0.001.

Since the rather unspecific NFκB- and MAP kinase-inhibitor dexamethason dose-dependently inhibited all cytokine production induced by stimulation of mDCs with rFlaA:Betv1, we investigated the contribution of MAP kinase-signaling to rFlaA:Betv1-induced activation of mDC metabolism and production of pro- and anti-inflammatory cytokines by applying MAPK-specific inhibitors (Figure 4 and Figure 5). For this we pre-treated the mDCs with either SP600125 (JNK MAPK inhibitor), U0126 (MEK1/2 MAPK inhibitor) or SB202190 (p38α/β MAPK inhibitor).

Here, only pre-treatment with the JNK MAPK inhibitor SP600125 but not the p42/44 MAPK inhibitor U0126 or the p38 MAPK inhibitor SB202190 dose-dependently resulted in a partial inhibition of the rFlaA:Betv1-induced Warburg effect (by 23.5% for the highest concentration of 25 µM SP600125), glucose consumption, and metabolic rate (Figure 4). Pre-treatment with SP600125 also inhibited rFlaA:Betv1-induced IL-10, IL-6, and TNF-α production, while enhancing both IL-1β and IL-12p70 production (Figure 5).

The MEK1/2 inhibitor U0126 dose-dependently suppressed rFlaA:Betv1-induced IL-10, IL-1β, and IL-6 secretion while having no effect on IL-12p70 and TNF-α secretion (Figure 5). Finally, the p38α/β MAPK inhibitor SB202190 dose-dependently inhibited FlaA:Betv1-induced IL-10 and TNF-α secretion, and boosted rFlaA:Betv1-induced IL-1β, as well as IL-6 and IL-12p70 secretion in the lower concentration range (Figure 5). 

Taken together, these data provide evidence that MAPK-signaling contributes to the induction of both pro- and anti-inflammatory cytokine secretion by rFlaA:Betv1 while only JNK MAPK-signaling is involved in rFlaA:Betv1-mediated activation of mDC metabolism.

**Figure 6:**
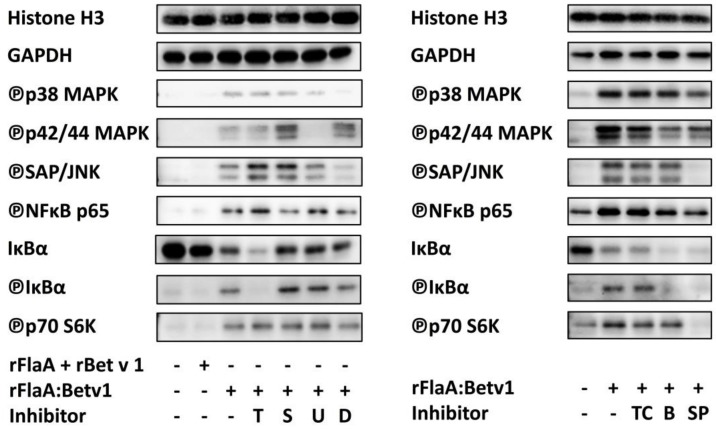
Both MAPK- and NFκB-signaling contribute to rFlaA:Betv1-induced mDC activation while only JNK MAPK-signaling contributes to mTOR-dependent phosphorylation of p70 S6 kinase: 1 × 10^6^ BALB/c mDCs were pre-treated for 3 h with triptolide (T, 10 nM), SB202190 (S, 60 µM), U0126 (U, 10 µg/mL), dexamethason (D, 40 ng/mL), TPCA-1 (TC, 60 nM), BMS-345541 (B, 1 µM), or SP600125 (SP, 25 µM) washed, and subsequently stimulated for 30 min with the indicated proteins. Target proteins in lysates were detected by Western Blot (Table 1). Data are representative or mean results of two to five independent experiments ± SD.

In line with these results, supporting a contribution of both NFκB- and MAPK-signaling to rFlaA:Betv1-mediated mDC activation, stimulation of mDCs with rFlaA:Betv1, but not with rFlaA + rBet v 1, resulted in an increased phosphorylation of p38 MAPK, p42/44 MAPK, SAP/JNK, NFκB p65, and IκBα as well as reduced levels of non-phosphorylated IκBα (Figure 6). 

Here, pre-treatment with triptolide slightly increased SAP/JNK and NFκB phosphorylation and inhibited IκBα phosphorylation, but otherwise did not alter rFlaA:Betv1-induced MAPK-phosphorylation, while phosphorylation of p42/44 and SAP/JNK MAPKs and IκBα phosphorylation was slightly enhanced by pre-treatment with SB202190 (Figure 6). U0126 selectively abrogated p42/44 MAPK phosphorylation, while dexamethason selectively inhibited rFlaA:Betv1-induced p38 MAPK and SAP/JNK phosphorylation (Figure 6). 

Of the tested IκBα inhibitors only BMS-345541 pre-treatment reduced NFκBp65 phosphorylation while both BMS-345541 and TPCA-1 reduced p42/44 phosphorylation (Figure 6). Here, neither BMS-345541 nor TPCA-1 had any effect on rFlaA:Betv1-induced SAP/JNK and p38 phosphorylation (Figure 6). As expected, SP600125 pre-treatment abrogated rFlaA:Betv1-induced SAP/JNK phosphorylation while also reducing the phosphorylation of p38, p42/44, NFBp65, and IκBα (Figure 6). Of the tested inhibitors only SP600125 was sufficient to suppress rFlaA:Betv1-induced p70 S6 kinase phosphorylation (Figure 6).

## 4. Discussion

Fusion proteins containing the TLR5-ligand flagellin and different antigens have been repeatedly shown to be effective vaccines boosting immune responses against fused and otherwise often poorly immunogenic antigens [12,13,14,15,16,17,18,20,23]. Despite this combination of clinical efficacy and safety, the mechanisms by which such fusion proteins modulate immune responses are less well understood. 

Therefore, the aim of the present study was to characterize the signaling pathways contributing to the induction of pro- and anti-inflammatory cytokine secretion in mDCs stimulated with flagellin: antigen fusion proteins and their contribution to the observed activation of mDC metabolism.

**Figure 7:**
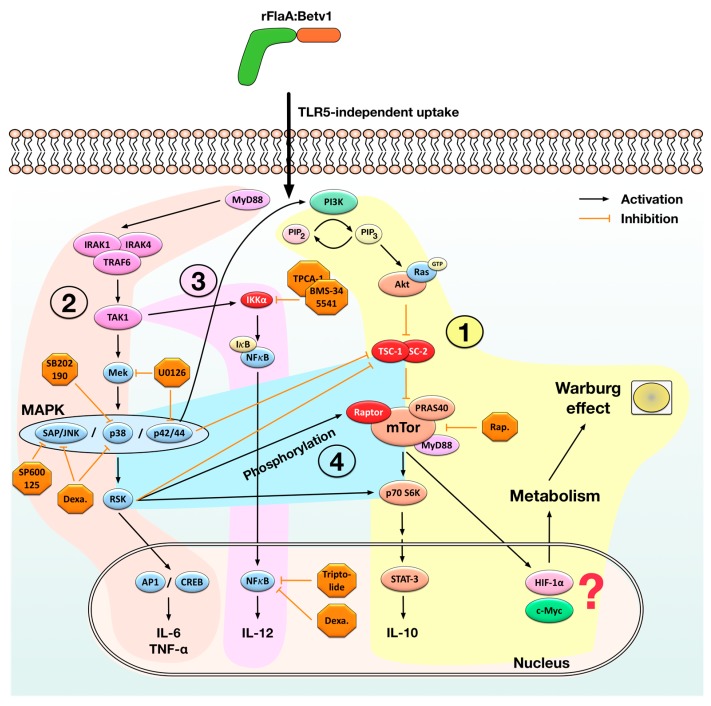
Suggested mechanism of rFlaA:Betv1-mediated mDC activation. Stimulation of mDCs with rFlaA:Betv1 results in enhanced uptake of the fusion protein and a mammalian target of rapamycin (complex 1) (mTORC1)-dependent activation of mDC metabolism and immune modulatory IL-10 secretion, likely mediated by hypoxia-inducible factor 1-alpha (HIF-1α)- and avian myelocytomatosis virus oncogene cellular homology (c-Myc)- and signal transducer/activator of transcription 3 (STAT-3)-signaling, respectively (1). rFlaA:Betv1 also triggers a myeloid differentiation primary response 88 (MyD88)-dependent, interleukin-1 receptor-associated kinase 1 (IRAK1)/IRAK4/TNF receptor associated factor 6 (TRAF6)/transforming growth factor beta-activated kinase 1 (TAK1)-mediated activation of the MAP kinase signaling pathway (2). Once activated, MAPK-signaling can cross-trigger activation and nuclear translocation of NFκB via TAK1-mediated activation of IKKα (3). Here, both NFκB- (promoting IL-12 secretion) and MAP kinase-signaling (promoting IL-6 and TNF-α secretion) contribute to rFlaA:Betv1-induced pro-inflammatory cytokine secretion. In addition, the activation of MAP kinase signaling can cross-activate the mTOR pathway by either inhibiting tuberous sclerosis complex 1/2 (TSC-1/TSC-2) complexes, phosphorylation of regulatory associated protein of mTOR (RAPTOR), or direct activation of mTOR downstream targets like p70 S6 kinase by ERK/ribosomal S6 kinase (RSK) (4). The MAPK-dependent mTOR activation also contributes to IL-10 secretion while contributing to rFlaA:Betv1-induced activation of mDC metabolism via JNK MAPK activation. The different inhibitors used in this study are indicated in orange, orange arrows depict inhibitory signals, black arrows depict activating signals.

We recently reported the mechanism of IL-10 induction by a fusion protein consisting of flagellin A from *Listeria monocytogenes* and the major birch pollen allergen Bet v 1 (rFlaA:Betv1) [22]: We observed, that stimulation of mDCs with rFlaA:Betv1, but not the mixture of rFlaA + rBet v 1, resulted in a TLR5-independent but MyD88-dependent activation of the mTOR complex. This activation of mTOR was likely mediated by the PIP3-mediated activation of protein kinase B (Akt) removing tuberous sclerosis-1/2 complex (TSC-1/2)-mediated suppression of mTOR activation (Figure 7). rFlaA:Betv1-mediated mTOR activation resulted in rapamycin-sensitive phosphorylation of the downstream p70 S6 kinase, activation of mDC metabolism, and production of the immunosuppressive cytokine IL-10 (Figure 7) [22]. However, the signaling pathways contributing to pro-inflammatory cytokine secretion induced by the fusion protein remained unclear.

Experiments using inhibitors of both MAPK- and NFκB-signaling (toxic effects of the used inhibitors were excluded by viability staining, Repository Figure S1) revealed that both signaling pathways contribute to cytokine secretion induced by the fusion protein. Our data support the hypothesis, that in mDCs rFlaA:Betv1 triggers a classical MyD88-dependent, IRAK1/IRAK4/TRAF6/TAK1-mediated activation of MAP kinase signaling (Figure 7). Indeed, Western Blot analysis confirmed the activation of SAP/JNK, p38, and p42/p44 MAPK upon stimulation of mDCs with rFlaA:Betv1 likely resulting in the subsequent production of pro-inflammatory cytokines via p42/44/ribosomal S6 kinase (RSK)-mediated activation of the transcription factors activator protein 1 (AP1) and cAMP response-binding element (CREB) (Figure 7). 

Here, in line with the available literature for other TLR-ligands [36], inhibition of p42/44 MAPK phosphorylation by pre-treatment with U0126 dose-dependently suppressed rFlaA:Betv1-induced IL-6 and IL-10 secretion while having no effect on IL-1β, IL-12p70, or TNF-α production. Similar results (suppression of IL-10, IL-6, and TNF-α secretion) were observed upon pre-treatment with the p38α and p38β MAPK inhibitor SB202190 (although in most cases not reaching statistical significance). Interestingly, JNK MAPK inhibition by pre-treatment of mDCs with SP600125 not only inhibited rFlaA:Betv1-induced IL-10, IL-6, and TNF-α secretion, but also partly suppressed the activation of mDC metabolism (Figure 4 and Figure 5). Therefore, the results obtained with the different MAPK inhibitors suggest that activation of p38-, p42/44-, and JNK MAP kinases contributes to rFlaA:Betv1-induced IL-10, IL-6, and TNF-α secretion while only activation of the JNK MAPK contributes to the activation of mDC metabolism. 

Once activated, the intracellular cascade leading to the activation of MAPK-signaling can also cross-trigger activation and nuclear translocation of NFκB via TAK1-mediated activation of IKKα (Figure 7). In line with this, stimulation of mDCs with rFlaA:Betv1 resulted in an increased phosphorylation of both NFκB p65 and IκBα while levels of non-phosphorylated IκBα were reduced 30 min post stimulation compared to either unstimulated or rFlaA + rBet v 1-stimulated mDCs (Figure 6). 

Mechanistically, inhibition of NFκB-signaling using the rather broadly acting inhibitors dexamethason, which also inhibited p38 MAPK and SAP/JNK MAPK phosphorylation (Figure 6), while triptolide inhibited both rFlaA:Betv1-induced pro- and anti-inflammatory cytokine secretion without suppressing phosphorylation of the mTOR target protein p70 S6 kinase and the activation of mDC metabolism (Figure 2, Figure 3 and Figure 6). In accordance with these results, dexamethason was reported to represses pro-inflammatory gene expression via inhibition of NFκB- and MAPK-activation during TLR engagement [37] while triptolide has more unspecific effects, interfering with a number of transcription factors including NFκB [38] as well as p53 [39], nuclear factor of activated T-cells (NFAT) [38], and heat shock factor protein 1 (HSF-1) [40].

Here, inhibition of NFκB-signaling by pre-treatment with the IKK-β inhibitors BMS-345541 or TPCA-1 had no influence on either mDC metabolism (Figure 2), IL-6, or IL-10 secretion but does-dependently inhibited IL-12 secretion while enhancing IL-1β production (Figure 3). In addition, only BMS-345541 pre-treatment was shown to suppress rFlaA:Betv1-induced TNF-α secretion (Figure 3). 

Of note, pre-treatment of the mDCs with BAY-11, which irreversibly inhibits non-canonical, TNF-α-induced IκB-α phosphorylation had no effect on rFlaA:Betv1-induced cytokine secretion and activation of mDC metabolism. This result suggests, that for the investigated fusion protein autocrine, TNF-α-induced IκB-α phosphorylation does not contribute to the observed mDC activation. 

Recent findings have suggested that ribosomal proteins may also control NFκB activity: the ribosomal protein S3 was found to interact with IκBα in resting HEK293 cells thereby sequestering p65 from the NFκB complex [41] and the ribosomal protein rpL3 was reported to reduce NFκB activity by increasing the stability of IκBα in Calu-6 cells [42]. In light of these reports, a possible interaction of either rFlaA:Betv1 or other proteins induced by stimulation of mDCs with rFlaA:Betv1 with cellular ribosomal proteins may also contribute to rFlaA:Betv1-mediated NFκB activation.

In summary, the results obtained with the different inhibitors of NFκB-signaling suggest the observed NFκB activation (Figure 6) to promote rFlaA:Betv1-induced IL-12 production while having only minor contributions to both the secretion of the other cytokines and activation of mDC metabolism.

Interestingly, of the tested inhibitors only the JNK inhibitor SP600125 was sufficient to prevent the mTOR-dependent [22] phosphorylation of p70 S6 kinase (Figure 6). Therefore, so far the obtained results suggest, the activation of both NFκB- and MAPK-signaling to contribute to rFlaA:Betv1-mediated cytokine secretion (NFκB: IL-12 and MAPK: IL-10, IL-6, TNF-α) while only JNK MAPK activation contributes to metabolic activation. 

Since the rFlaA:Betv1-induced, mTOR-dependent IL-10 secretion was also inhibited by MAPK-inhibitors, these results furthermore suggest an interaction between rFlaA:Betv1-induced MAPK-activation and the activation of the mTOR pathway by the rFlaA:Betv1 fusion protein. Here, the data support that the activation of both NFκB- and MAPK-signaling upon stimulation of mDCs with rFlaA:Betv1 is located upstream of the recently published activation of the mTOR1-complex [22]. 

Indeed, there is extensive evidence that activated p42/44 MAPK-signaling can cross-activate mTOR-signaling by activating phosphatidylinositol 3 kinase (PI3K) [43,44,45,46] (Figure 6). Moreover, activation of p42/44/RSK-signaling can lead to mTOR-activation by (1) phosphorylation of TSC-2, which releases TSC-1/2-mediated mTOR inhibition [47,48], (2) phosphorylation of RAPTOR (regulatory associated protein of mTOR) which promotes mTORC1-activation, and (3) direct activation of the mTOR downstream target protein p70 S6 kinase [49,50,51] (Figure 7). Therefore, the inhibition of rFlaA:Betv1-induced IL-10 secretion by SB202190 may also be explained by its reported capacity to induce cross-inhibition of the PI3K/mTOR pathway [52].

TLR-stimulation was described to induce a rigorous glycolytic phenotype in macrophages, which may completely abolish oxidative phosphorylation favoring the generation of lactate-derived energy from glucose in a process termed the Warburg effect [53,54]. In immune cells, this increased glucose consumption sustains essential immune-related functions such as cytokine production, phagocytosis, ROS generation, and proliferation [55]. Interestingly, the induced Warburg effect and the associated increase in cellular glucose metabolism were independent of both p38 and p42/44 MAPK- and NFκB-dependent cytokine secretion, but partially dependent on rFlaA:Betv1-mediated JNK activation. These results suggest, that the activation of mDC metabolism is either initiated earlier than the observed cytokine secretion or is controlled by different pathways that are activated in parallel and are, with the exception of JNK-signaling, independent of NFκB- and MAPK-signaling.

Activation of the mTOR pathway, e.g. by elevated levels of succinate, reactive oxygen species (ROS), or TLR-stimulation [56,57,58] drives glycolytic metabolism by inducing two central transcription factors: Hypoxia-inducible factor 1-alpha (HIF1α) and avian myelocytomatosis virus oncogene cellular homology (c-Myc) [59,60]. Whereas HIF1α, usually stabilized during anaerobic/hypoxic conditions, does not support mitochondrial respiration via oxidative phosphorylation [61,62], c-Myc promotes both glycolytic gene expression and mitochondrial respiration [63,64]. Moreover, mTOR was shown to phosphorylate STAT3 at Ser727 likely driving IL-10 secretion [65,66]. Therefore, the results obtained in this study suggest that the events downstream of the observed mTOR activation (which was shown to be independent of p38 and p42/44 MAPK and NFκB activation) namely rFlaA:Betv1-induced IL-10 secretion and the Warburg effect are regulated by different factors (STAT-3 possibly promoting IL-10 secretion, HIF-1α and c-Myc possibly promoting the activation of glucose metabolism). We summarized our current understanding of the molecular signaling events contributing to rFlaA:Betv1-mediated mDC activation in Figure 7. 

In summary, we showed that both NFκB- and MAPK-signaling contribute to rFlaA:Betv1-mediated cytokine secretion while only JNK MAPK activation contributes to the metabolic activation in murine mDCs in vitro. Better understanding of the mechanisms by which these promising vaccine candidates modulate immune responses will improve their safety and efficacy in clinical settings.

## Figures and Tables

**Table 1:** Quantification of Western Blot results. Quantification of both band intensities shown in Figure 6 as well as the confirmatory experimental repeats (data not shown) was performed using ImageJ and RLU values were normalized to the unstimulated samples of each experiment. Data are mean results of two to three independent experiments ± SD.

	Histon H3	GAPDH	℗p38	℗p42/44	℗SAP/JNK	℗NFκBp65	IκB-α	℗IκB-α	℗p70 S6K
**rFlaA + rBet v 1**	1.06 ± 0.02	0.95 ± 0.16	1.07 ± 0.21	0.83 ± 0.16	1.68 ± 1.35	5.01 ± 5.45	0.79 ± 0.07	0.71 ± 0.15	0.71 ± 0.31
**rFlaA:Betv1**	0.99 ± 0.07	1.09 ± 0.22	2.64 ± 1.19	3.57 ± 0.38	2.81 ± 0.22	16.79 ± 6.81	0.44 ± 0.02	1.52 ± 0.17	2.07 ± 0.85
**rFlaA:Betv1 + dexamethason**	1.17 ± 0.30	0.83 ± 0.16	0.65 ± 0.74	9.51 ± 6.49	1.97 ± 0.27	34.02 ± 32.76	0.37 ± 0.05	2.11 ± 0.40	3.64 ± 1.43
**rFlaA:Betv1 + TPCA-1**	0.92 ± 0.04	1.05 ± 0.11	2.62 ± 1.01.	2.71 ± 0.86	2.82 ± 0.52	2.30 ± 0.70	0.43 ± 0.05	1.46 ± 0.03	1.37 ± 0.15
**rFlaA:Betv1 + BMS-345541**	0.88 ± 0.06	1.04 ± 0.23	2.57 ± 0.94	1.86 ± 0.57	2.97 ± 0.68	1.98 ± 0.69	0.29 ± 0.10	0.78 ± 0.16	1.57 ± 0.33
**rFlaA:Betv1 + triptolide**	1.17 ± 0.08	1.05 ± 0.24	3.87 ± 2.38	11.42 ± 12.43	5.79 ± 2.38	32.03 ± 20.29	0.18 ± 0.03	1.03 ± 0.18	4.99 ± 2.52
**rFlaA:Betv1 + Sp600125**	0.92 ± 0.10	1.07 ± 0.12	1.96 ± 0.78	2.65 ± 1.20	1.14 ± 0.34	1.93 ± 0.60	0.31 ± 0.06	0.67 ± 0.25	0.74 ± 0.32
**rFlaA:Betv1 + U0126**	1.16 ± 0.22	0.96 ± 0.29	2.66 ± 0.47	1.31 ± 0.83	4.28 ± 0.22	32.28 ± 23.97	0.42 ± 0.15	2.47 ± 0.08	4.55 ± 1.65
**rFlaA:Betv1 + SB202190**	1.10 ± 0.11	0.93 ± 0.29	3.40 ± 1.54	15.80 ± 14.57	5.07 ± 2.71	38.11 ± 29.42	0.43 ± 0.15	2.46 ± 0.40	5.18 ± 2.81

## Supplementary Materials

The following are available online at https://www.mdpi.com/2073-4409/8/4/355/s1, Figure S1: Toxicity of the different inhibitors on mDC cultures, Figure S2: Effect of triptolide pre-treatment on rFlaA:Betv1-induced cell metabolism and cytokine secretion, Figure S3: TNF-α-induced IkB-α phosphorylation does not contribute to rFlaA:Betv1-induced cell metabolism and cytokine secretion.

## Abbreviations

AP1	Activator protein 1
APC	Antigen presenting cell
Akt	Protein kinase B
c-Myc	Avian myelocytomatosis virus oncogene cellular homology
ConA	Concanavalin A
CREB	cAMP response element-binding protein
DC	Dendritic cell
HIF1α	Hypoxia-inducible factor 1-alpha
IFN-γ	Interferon γ
Ig	Immunoglobulin
IκB	Inhibitor of kappa B
IL	Interleukin
IRAK	Interleukin-1 receptor-associated kinase
LPS	Lipopolysaccharide
Mal/TIRAP	MyD88 adapter-like/toll-interleukin 1 receptor domain containing adaptor protein
MAP(K)	Mitogen-activated protein (kinase)
mDC	Myeloid dendritic cell
MEK1/2	MAP kinase/ERK kinase-1/2
mTOR(1-complex)	Mammalian target of rapamycin (complex 1)
MyD88	Myeloid differentiation primary response 88
NFAT	Nuclear factor of activated T cells
NFκB	Nuclear factor “kapa-light-enhancer” of activated B cells
OD	Optical density
PBMC	Peripheral blood mononuclear cells
PI3K	Phosphatidylinositol 3-kinase
PRR	Pattern recognition receptor
RAPTOR	Regulatory associated protein of mTOR
Ras-ERK	Ras-extracellular signal-regulated kinase
rBet v 1	Betula verucosa allergen 1
rFlaA	Recombinant flagellin A
rFlaA:Betv1	Recombinant fusion protein consisting of FlaA and Bet v 1
Rel(A)	Reticuloendotheliosis oncogene cellular homolog (A)
RSK	ERK/ribosomal S6 kinase
STAT-3	Signal transducer and activator of transcription 3
TAK1	Transforming growth factor beta-activated kinase 1
TC	T cell
TNF-α	Tumor necrosis factor α
TH1/2	T helper type 1/2
TLR(5)	“Toll”-like receptor (5)
TRAF6	TNF receptor associated factor 6
TRAM/TICAM-2	TRIF-related adaptor molecule/ TIR domain-containing adapter molecule 2
TRIF/TICAM-1	TIR-domain-containing adapter-inducing interferon-β/ TIR domain-containing adapter molecule 1
TSC-1/2	Tuberous sclerosis complex ½
