# Supplementary material for: NFκB- and MAP-Kinase Signaling Contribute to the Activation of Murine Myeloid Dendritic Cells by a Flagellin A: Allergen Fusion Protein
Source: Cells. 2019 Apr 15;8(4):355. doi: 10.3390/cells8040355 (PMC6523117; doi:10.3390/cells8040355)
Supplement: Supplementary file 1 [file cells-08-00355-s001.pdf]

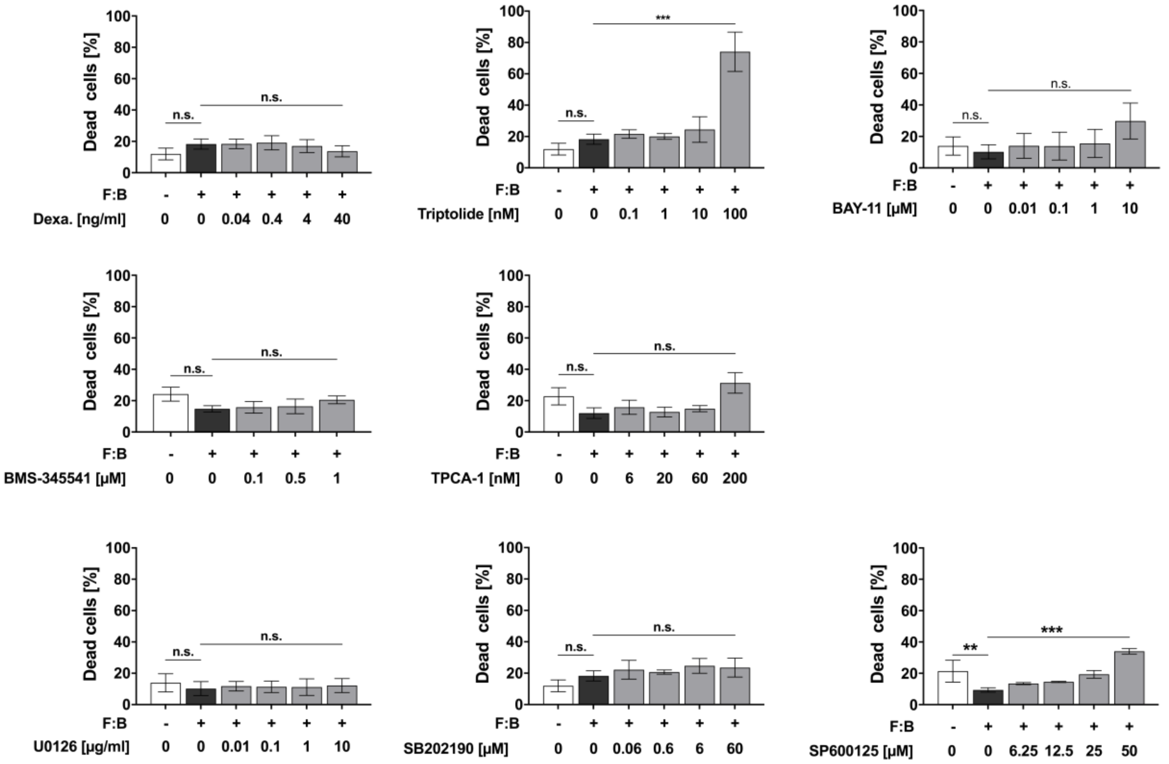


**Repository Figure S1: Toxicity of the different inhibitors on mDC cultures.** 0.5x10[6] BALB/c mDCs were pre-treated with the indicated inhibitor concentrations for 90 min and subsequently stimulated with 27.4 µg rFlaA:Betv1 for another 24 h. The frequency of dead cells in mDC cultures 24 h post-stimulation with rFlaA:Betv1 was determined using the fixable viability dye eFlour780. Inhibitor concentrations showing toxic effects were excluded from the subsequent analysis. Data are mean results from three independent experiments±SD.

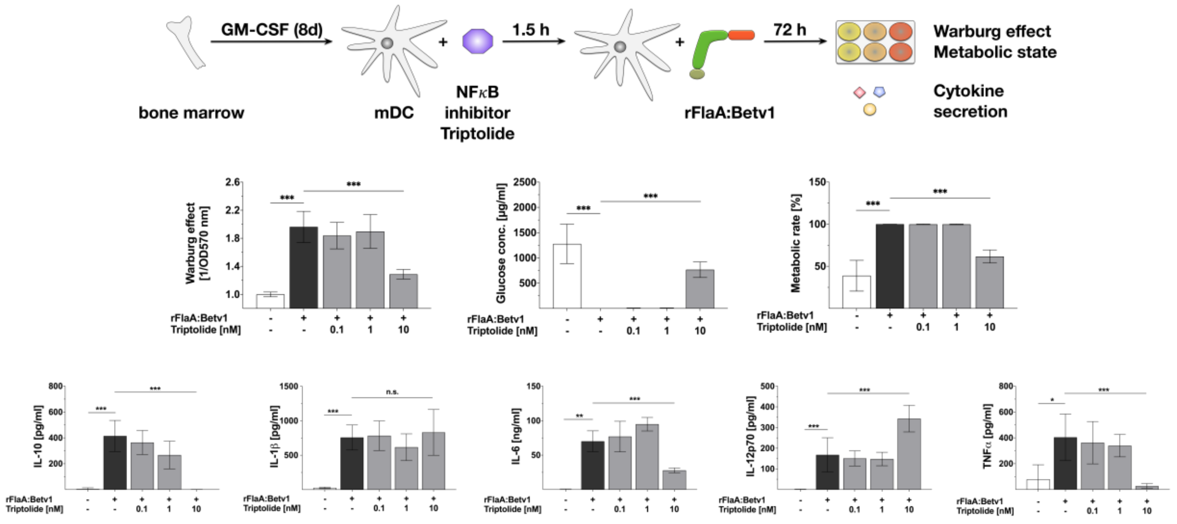


**Repository Figure S2: Effect of triptolide pre-treatment on rFlaA:Betv1-induced cell metabolism and cytokine secretion.** 0.5x10[6] BALB/c mDCs were pre-treated with the indicated concentrations of the NFκB inhibitor triptolide for 90 min and subsequently stimulated with 27.4 µg rFlaA:Betv1 for another 72 h. The metabolic state

of the stimulated mDCs was determined 72 h post-stimulation. Cytokine secretion into cell supernatants was determined 72 h post-stimulation by ELISA. Data are mean results of three independent experiments±SD.

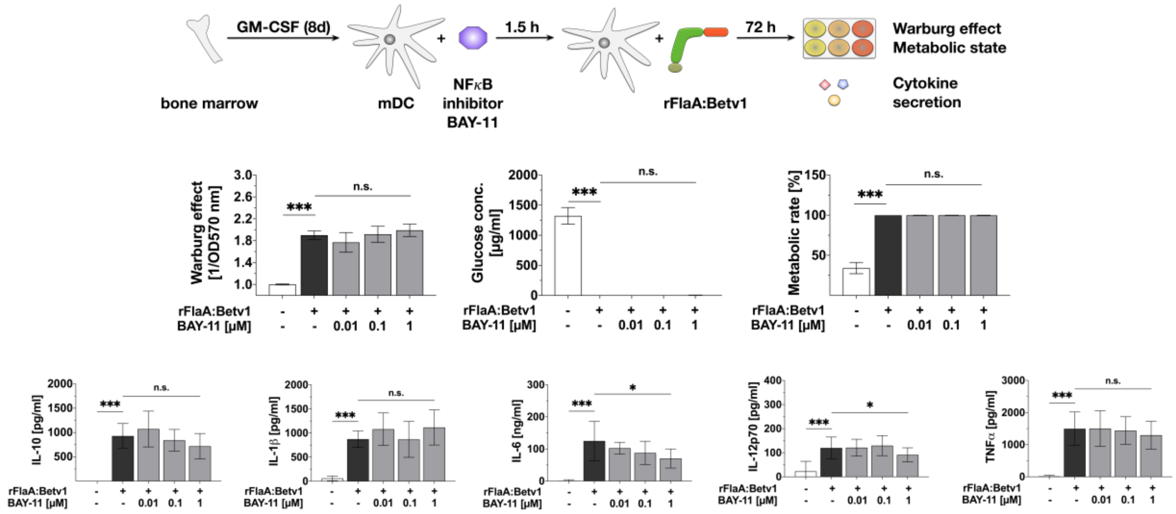


**Repository Figure S3: TNF-$\alpha$-induced IkB-$\alpha$ phosphorylation does not contribute to rFlaA:Betv1-induced cell metabolism and cytokine secretion.** 0.5x10$^6$ BALB/c mDCs were pre-treated with the indicated concentrations of the irreversible inhibitor of TNF-$\alpha$-induced IkB-$\alpha$ phosphorylation BAY-11-7082 for 90 min and subsequently stimulated with 27.4 µg rFlaA:Betv1 for another 72 h. The metabolic state of the stimulated mDCs was determined 72 h post-stimulation. Cytokine secretion into cell supernatants was determined 72 h post-stimulation by ELISA. Data are mean results of three independent experiments±SD.
